# Structural Basis for Antigen Recognition by Transglutaminase 2-specific Autoantibodies in Celiac Disease*

**DOI:** 10.1074/jbc.M115.669895

**Published:** 2015-07-09

**Authors:** Xi Chen, Kathrin Hnida, Melissa Ann Graewert, Jan Terje Andersen, Rasmus Iversen, Anne Tuukkanen, Dmitri Svergun, Ludvig M. Sollid

**Affiliations:** From the ‡ Centre for Immune Regulation and Department of Immunology, University of Oslo and Oslo University Hospital, N-0372 Oslo, Norway and; § European Molecular Biology Laboratory, Hamburg Outstation, D-22607 Hamburg, Germany

**Keywords:** antibody, epitope mapping, mutagenesis, small-angle x-ray scattering (SAXS), surface plasmon resonance (SPR), transglutaminase, celiac disease

## Abstract

Antibodies to the autoantigen transglutaminase 2 (TG2) are a hallmark of celiac disease. We have studied the interaction between TG2 and an anti-TG2 antibody (679-14-E06) derived from a single gut IgA plasma cell of a celiac disease patient. The antibody recognizes one of four identified epitopes targeted by antibodies of plasma cells of the disease lesion. The binding interface was identified by small angle x-ray scattering, *ab initio* and rigid body modeling using the known crystal structure of TG2 and the crystal structure of the antibody Fab fragment, which was solved at 2.4 Å resolution. The result was confirmed by testing binding of the antibody to TG2 mutants by ELISA and surface plasmon resonance. TG2 residues Arg-116 and His-134 were identified to be critical for binding of 679-14-E06 as well as other epitope 1 antibodies. In contrast, antibodies directed toward the two other main epitopes (epitopes 2 and 3) were not affected by these mutations. Molecular dynamics simulations suggest interactions of 679-14-E06 with the N-terminal domain of TG2 via the CDR2 and CDR3 loops of the heavy chain and the CDR2 loop of the light chain. In addition there were contacts of the framework 3 region of the heavy chain with the catalytic domain of TG2. The results provide an explanation for the biased usage of certain heavy and light chain gene segments by epitope 1-specific antibodies in celiac disease.

## Teaser

**Background:** Knowledge of how celiac disease autoantibodies recognize transglutaminase 2 (TG2) is limited.

**Results:** The interaction between TG2 and a celiac disease epitope 1 anti-TG2 antibody was studied by small angle x-ray scattering and mutational analysis.

**Conclusion:** TG2 residues Arg-116 and His-134 are part of epitope 1.

**Significance:** The study gives insights into key aspects of celiac disease.

## Introduction

Celiac disease is an inflammatory enteropathy characterized by a harmful immune response to dietary gluten antigen (1). Patients with active disease have autoantibodies to the enzyme transglutaminase 2 (TG2)^4^ of various isotypes in the blood (2), and IgA- and IgM- producing plasma cells specific for TG2 are abundantly present in small intestinal disease lesions (3). Testing for serum IgA anti-TG2 antibodies is central in the diagnostic workup of the disease (4). The production of anti-TG2 autoantibodies is contingent on dietary exposure to gluten as the antibodies disappear from serum (5, 6), and the number of TG2-specific plasma cells in the gut mucosa drops when patients commence a gluten-free diet (3). TG2 is involved in celiac disease not only as the target of autoantibodies. The enzyme also plays an important role in creating immunogenic, deamidated epitopes of gluten that are recognized by CD4 T cells in the context of celiac disease-associated HLA-DQ molecules (7). It is likely that the dual involvement of TG2 in celiac disease, as a generator of T-cell epitopes and as a target for autoantibodies, is causally linked, although the mechanism for this has not been settled (8).

TG2 is a multifunctional enzyme involved in the cellular processes of apoptosis (9), adhesion (10), and extracellular matrix modification (11). A major function of TG2 is to catalyze calcium-dependent transamidation and deamidation reactions. The enzyme targets polypeptide glutamine residues in a sequence-specific manner and either cross-links them to a primary amine, which can be a lysine residue of another polypeptide (transamidation), or converts them to glutamate through a reaction with water (deamidation). TG2 can also have other functions such as GTPase/G-protein, kinase, and disulfide isomerase (12). The structure and function of TG2 are influenced not only by calcium but also by nucleotide phosphates (13). Crystal structures of TG2 with bound GDP (14) (PDB code 1KV3), GTP (15) (PDB code 4PYG), or ATP (16) (PDB code 3LY6) have demonstrated that these forms of TG2 adopt a “closed” conformation, whereas TG2 with the active site occupied by an inhibitory gluten peptide mimic (17) (PDB code 2Q3Z) or other similar inhibitors (PDB codes 3S3P, 3S3S, and 3S3J) adopts an “open” conformation. In the open conformation the four domains of TG2 are arranged in an extended configuration, whereas in the closed conformation the two C-terminal domains are folded in on the catalytic core domain. The N-terminal domain only shows minor structural changes between the two different conformations.

There is limited knowledge about the interaction of TG2 autoantibodies with TG2. A recent study with polyclonal sera of celiac disease patients indicated that there is an important conformational epitope involving residues Arg-19, Glu-153, and Met-659 located in three different domains of TG2 (18). Studies of a panel of TG2-specific monoclonal antibodies derived from single plasma cells of celiac lesions (3) indicated the existence of four common epitopes (epitope 1–4) that are conformational and clustered in the N-terminal part of the TG2 molecule (19). The epitopes were found to correlate with the VH usage of the antibodies; epitope 1 antibodies mainly used *IGHV5* gene segments, epitope 2 antibodies used *IGHV3* gene segments, and epitope 3 antibodies mainly used *IGHV4* gene segments (19). Epitope 1 is a major epitope as 30 of 57 monoclonal antibodies derived from single TG2-reactive plasma cells were found to be epitope 1-specific (19). By hydrogen/deuterium exchange and subsequent mutational analysis of TG2 it was recently demonstrated that residues Lys-30 and Glu-8 are part of epitope 1, whereas residue Arg-19 is part of epitope 2 (20).

To further explore the structural basis for antigen recognition by anti-TG2 autoantibodies, we studied in detail the interaction of a prototype epitope 1 monoclonal antibody (679-14-E06) with TG2. Despite intensive effort, co-crystallization trials of the Fab fragment of 679-14-E06 with various forms of TG2 were unsuccessful. However, we succeeded in solving the structure of the antibody Fab fragment by x-ray crystallography, and we studied the interaction of the Fab fragment with TG2-GDP by small angle x-ray scattering (SAXS). The interaction site predicted by the SAXS analysis was validated through generation of single amino acid TG2 mutants that were then tested for interaction with 679-14-E06 by surface plasmon resonance (SPR) as well as in ELISA using a panel of 38 other celiac disease TG2-specific monoclonal antibodies. Moreover, molecular dynamics (MD) simulations were performed to investigate the binding mechanism in greater detail. The results provide novel information about epitope 1 of TG2 and the key residues recognized by autoantibodies of celiac disease patients.

## Experimental Procedures

### 

#### 

##### Production of Anti-TG2 Autoantibodies and Fab Fragment

Anti-TG2 autoantibodies were cloned and expressed as human IgG1 as previously described (3). The Fab fragment of antibody 679-14-E06 was generated by adding a stop codon in the heavy chain gene after residue 231 (^228^PKSC^231^) by PCR using the forward primer 5′-TTTCTAGTAGCAACTGCAAC-3′ and the reverse primer 5′-GAAAGTTGAGCCCAAATCTTGTTGAAGCTTGGAT-3′ followed by subcloning into the expression vector between the AgeI and HindIII restriction sites. Antibodies in which the heavy or light chain of 679-14-E06 was swapped with the heavy or light chain of the non-TG2 reactive antibody 679-14-A04 were also generated. 679-14-E06 carries *IGHV5-51* and *IGKV1-5*, whereas 679-14-A04 carries *IGHV3-49* and *IGKV4-1*. Plasmids encoding the heavy and light chains were co-transfected into HEK 293F cells by using 293fectin (Invitrogen) or polyethyleneimine (Polysciences Inc). HEK 293F cells were cultured at 37 °C with shaking for 6 days. Anti-TG2 autoantibodies were purified from culture supernatants by affinity chromatography using a protein G column (GE Healthcare), whereas the Fab fragment was purified using a protein L column (GE Healthcare). The purity of the proteins was confirmed by SDS-PAGE.

##### Crystallization, Structure Determination and Refinement of the 679-14-E06 Fab Fragment

The Fab fragment of 679-14-E06 was concentrated to 8.82 mg/ml, and crystallization screening was set up using a robot (Douglas instrument). Two kits, Crystal Screen^TM^ and Crystal Screen 2^TM^ (Hampton Research), were used for screening. Clusters of crystals were obtained after 1 week with the condition 0.01 m nickel (II) chloride hexahydrate, 0.1 m Tris, pH 8.5, 20% w/v polyethylene glycol monomethyl ether 2000. Microseeding was applied to optimize the Fab fragment to form single crystals.

A single crystal was transferred to cryosolution consisting of the crystallization buffer and 25% glycerol. The soaked crystal was flash-frozen in liquid nitrogen. A complete data set was collected on the BM30 beam at European Synchrotron Radiation Facility, Grenoble, France. Data were processed and scaled with MOSFLM (21) and Aimless (22). The structure of the Fab fragment of 679-14-E06 was solved by molecular replacement using Phaser (23). Initially, an immunoglobulin heavy chain (PDB code 4HPO) and a light chain (PDB code 1DFB) were used as search models without success. Then the variable and constant domains of the heavy and light chains of these structures were used as separate templates, and a solution could be obtained. Several rounds of manual building by COOT (24) and refinement by REFMAC (25) and PHENIX (26) were carried out. The electron density map for the Fab was generally of high quality except for parts of the C region domains where the electron density was missing for residues 142–149, 198–207, and 225–231 of the heavy chain and 172–175 and 228–234 of the light chain. The amino acid numbering and definition of framework and complementary determining regions (CDR) was done according to the IMGT (International ImMunoGeneTics) convention (27). Structural visualization and generation of graphic illustrations were done with PyMOL.

##### Production and Purification of TG2 and TG2 Mutants

WT and mutant TG2 were produced as described previously (20, 28). Mutations were introduced with the QuikChange site-directed mutagenesis kit (Stratagene) or by PCR amplification followed by subcloning into the pET-28a vector (Novagen) between the NdeI and HindIII restriction sites. Correct sequences of all mutants were verified by DNA sequencing and protein sequence verification by mass spectrometry. The proteins were purified by nickel nitrilotriacetic acid affinity chromatography. A fraction of each protein was buffer-exchanged into HBS-EP buffer (0.01 m HEPES, 0.15 m NaCl, 2 mm EDTA 0.05% surfactant P20, pH 7.4) for SPR binding studies. The TG2 mutant K30E was not stable in this buffer and formed aggregates. Hence it was excluded from the SPR analysis. TG2 molecules used for complex formation with Fab fragment were further purified by anion exchange chromatography using a Mono Q column (GE Healthcare).

##### TG2-GDP Fab Fragment Complex Formation and Purification

Purified TG2 and the 679-14-E06 Fab fragment were mixed at a 1:2 molar ratio and incubated with 1 mm GDP for 2 h. The complex between TG2-GDP and the Fab fragment was purified by gel filtration on a Superdex200 10/300 GL column (GE Healthcare) in 20 mm Tris, pH 7.2, 150 mm NaCl, and 1 mm EDTA and eluted as a single symmetric peak. GDP was added to the sample to a final concentration of 1 mm before use. High purity of the complex was confirmed by SDS-PAGE and native PAGE. Native PAGE analysis further revealed very little dissociation, suggesting that the complex was stable and homogenous. In comparison, the complex with the open conformation of TG2 was less stable and thus was excluded from further analysis.

Additional testing of the complex stability was done by limited proteolysis. The purified complex of TG2-GDP/679-14-E06 Fab fragment (25 μg) was mixed with trypsin or chymotrypsin at a 1:500 (protease:complex) (w/w) ratio. The mixture was incubated on ice, and samples were taken out after 2, 15, 30, and 60 min. Proteolysis was stopped by incubation in SDS loading buffer at 96 °C for 10 min before the samples were analyzed by SDS-PAGE. Only a small degree of protein cleavage was observed, indicating that the complex had a compact and stable conformation, which allowed it to resist proteolysis.

##### Small Angle X-ray Scattering Data Collection and Modeling

SAXS data for TG2-GDP and 679-14-E06 Fab fragment as well as the complex of the two were collected at the SAXS beamline P12 at the PETRA III storage ring (Deutsches Elektronen-*S*ynchrotron, Hamburg) (29). Using a PILATUS 2M pixel detector at a sample-detector distance of 3.1 m and at an energy of 10 keV (λ = 1.24 Å), the range of momentum transfer 0.01 < s < 0.45 Å^−1^ was covered (s = 4π sinθ/λ, where 2θ is the scattering angle). For each construct, several solute concentrations in the range of ∼1–10 mg/ml were measured. Sample purity was verified with dynamic light scattering (DynaPro Nanostar) before the SAXS experiment. An automated sample changer was employed to load the samples and pump the sample through the observation capillary during the exposure period to constantly remove irradiated sample. For radial averaging, the *s* axis was calibrated with silver. Primary data processing steps were performed using the automated data pipeline SASFLOW (30).

SAXS analysis was performed using various programs of the ATSAS 2.6 package (30). The forward scattering *I*(0) and the radius of gyration *R_g_* were extracted from the Guinier approximation calculated with the AutoRG function within PRIMUS (31). These parameters were also computed from the entire scattering patterns using the indirect transform package GNOM (32), also providing the pair distribution function, *P*(*r*), of the particle and the maximum size *D*_max_. The molecular weight of the solute was evaluated by comparison of the forward scattering with that from a reference solution of bovine serum albumin (MW_monomer-dimer_ = 72; *I*(0)_BSA_ = 1797.8). The molecular weight estimations were cross-validated using the particle excluded (Porod) volumes as previously described (30). SAXS data from dilute monodisperse solutions can be used to generate low resolution three-dimensional structures without any prior knowledge on the size and shape of the molecule. Such so-called *ab initio* reconstructions were generated with the program DAMMIF (34). However, as the reconstruction of three-dimensional structures from SAXS data is inherently ambiguous further post-processing is required to assess the uniqueness of the models and check the stability of the solution. For this purpose, 10 independent DAMMIF runs were superimposed onto each other by SUPCOMB (35). The common structural features were determined using the program DAMAVER (36) to export a starting model for a final round of *ab initio* modeling by the program DAMMIN (37).

The theoretical scattering from the high resolution models of the Fab fragment of 679-14-E06 (PDB code 4ZD3) and TG2 with GTP (PDB code 4PYG) were calculated with the program CRYSOL (38) and compared with the respective scattering profiles. The x-ray structure of TG2 with GDP (PDB code 1KV3) misses electron density of some loops, which prevented further modeling. Therefore the x-ray structure of TG2 with GTP (PDB code 4PYG) was used. To improve the fit of the Fab fragment, missing portions were added with the program Coral (39). The program Oligomer (31) was employed for a better description of the experimental scattering profile of TG2. As solution scattering is sensitive to changes in the quaternary structure of macromolecules, it is particularly useful for the analysis of complexes. For example, a hybrid approach as implemented in the program SASREF (39) allows for modeling of the complex using solely the known structures of the individual subunits (rigid body modeling). For this purpose the high resolution models of the Fab fragment (PDB code 4ZD3) and TG2-GTP (PDB code 4PYG) were used to perform rigid body modeling with SASREF (39). First, the scattering amplitudes from the subunits are centered at the origin. Then rational and position parameters are determined to optimize the fit of the theoretical scattering curve from the resulting complex to the experimental data. Similar to the *ab initio* modeling, running rigid-body modeling several times can yield different models and should be done to indicate the ambiguity of the modeling. To define the footprint of the antibody binding to TG2, residues of TG2 that had any atoms <5.0 Å distance from any atom in the Fab fragment were identified using the CCP4 program CONTACT (40).

##### Antibody Binding to TG2 Measured in ELISA

ELISA assays using WT and mutant TG2 molecules as antigens were performed as described previously (20).

##### Surface Plasmon Resonance Analysis to Determine Antibody Binding Kinetics to TG2 Variants

SPR analyses were performed on a BIAcore 3000 instrument (GE Healthcare). Anti-TG2 autoantibodies were coupled to CM5 sensor chips using amine-coupling chemistry following the manufacturer's instructions. Each protein (2–4 μg/ml) was injected in 10 mm sodium acetate at pH 5.0 (GE Healthcare) to reach 700–2300 resonance units. Unreacted moieties on the CM5 surface were blocked with 1 m ethanolamine. In a control flow cell a non-TG2 reactive antibody (Influximab; Schering-Plough) was immobilized by the same procedure. Relative binding was assessed by injecting 500 nm WT or mutant TG2 molecules in HBS-EP buffer (0.01 m HEPES, 0.15 m NaCl, 2 mm EDTA, 0.05% surfactant P20, pH 7.4). Kinetic measurements were done by injecting serial dilutions (15.6/156–500/5000 nm) of TG2 molecules. All experiments were run with a flow rate of 40 μl/min at 25 °C. Binding data were zero-adjusted, and reference cell binding was subtracted. Kinetic rate constants were estimated using a simple Langmuir 1:1 ligand binding model provided by the BIAevaluation 4.1 software.

##### Molecular Dynamics Simulation

To obtain insights into the binding interface between TG2 and the Fab fragment, nanosecond time-scale molecular dynamics simulations were performed. MD simulations are well suited for studying protein-protein interactions at atomic level. The NAMD program was used for the simulations (41), and a CHARMM27 force field was employed for description of the protein (42). A TIP3P solvent model represented the water molecules (43). In addition, cavities inside proteins were detected and solvated according to an energy criterion using DOWSER (44). Constant particle number, constant pressure, and constant temperature (*NpT*) ensembles were assumed. Langevin dynamics was used to maintain constant temperature. Pressure was controlled using a hybrid Nose-Hoover Langevin piston method. An in-house computational pipeline for high throughput MD simulations and the visualization program VMD was used to prepare input files and to analyze the simulation trajectories with respect to the binding interface (45).

## Results

### 

#### 

##### Crystal Structure of the Fab Fragment of 679-14-E06

The final statistics of the refinement for the crystal structure of the Fab fragment of 679-14-E06 are listed in Table 1. The structure has been deposited in PDB with the PDB code 4ZD3. The Fab fragment displays a conventional immunoglobulin fold characterized by an anti-parallel β-sheet sandwich architecture with CDR loops forming the presumed antigen binding site. The electron density is well defined for most residues in the CDR loops except for Tyr-109 in the CDR-H3 and Tyr-110 and Ser-113 in the CDR-L3, reflecting the flexibility of the CDR3 loops.

**TABLE 1:** **Data collection and structure refinement statistics of the Fab fragment of antibody 679-14-E06** Values in parentheses are for the highest resolution shell.

**Data collection**	
Space group	P 1 21 1
Cell dimensions	
*a*, *b*, *c* (Å)	50.3400, 61.3800, 79.0500
α, β, γ (^o^)	90.0000, 100.9000, 90.0000
Resolution (Å)	49.53-2.40 (2.40-2.50)
*R*_sym_*^a^* or *R*_merge_	0.117 (0.605)
*I*/σ	9.3 (2.4)
Completeness (%)	99.2 (99.5)
Redundancy	3.4 (3.5)

**Refinement**	
Resolution (Å)	49.43-2.40
No. reflections	18,508
*R*_work_*^b^*/*R*_free_	20.04/26.38 (22.34/27.60)
No. atoms	
Protein	3097
Ligand/ion	
Water	78
B-factors	
Protein	40.40
Ligand/ion	
Water	31.13
Root mean square deviations	
Bond lengths (Å)	0.0092
Bond angles (°)	1.277
Ramachandran plot	
Core (%)	87.2
Allowed (%)	12.2
General (%)	0.3
Disallowed (%)	0.3

*^a^ R*_sym_ = Σ|*I*avg − Ii|/Σ*Ii*, where *Ii* is the observed intensity, and *I*avg is the average intensity of observations of symmetry-related reflections.

*^b^ R*_work_ = Σ|Fp − Fp(calc.)|/ΣFp, where Fp and Fp(calc.) are the observed and calculated structure factors; *R*_free_ is calculated with 5% of the data.

##### Small Angle X-ray Scattering Analysis of TG2-GDP, the 679-14-E06 Fab Fragment, and the Complex

**FIGURE 1.**
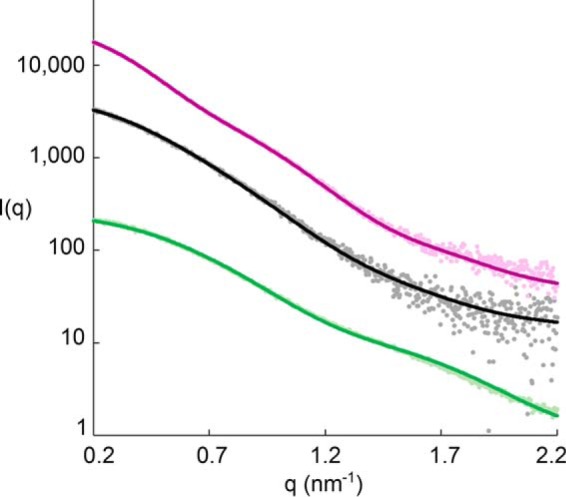
**Small angle x-ray scattering data.** Shown are scattering data and theoretical fits of the complex (*pink*), TG2-GDP (*gray*), and the Fab fragment (*green*). Shown are the scattering intensities *I*(*q*) as the functions of the scattering vector *q* (*q* = 4πsin(θ)/λ,where 2θ is the scattering angle, and λ is the wavelength). The profiles have been shifted along the *y* axis for better visualization.

To gain insights into the binding between a celiac disease antibody and TG2, we performed SAXS on the complex made up of the Fab fragment of antibody 679-14-E06 and TG2-GDP. In addition, we collected scattering data of the two subunits individually and used high resolution crystal structures of TG2-GTP (15) and the 679-14-E06 Fab fragment to evaluate the data. A series of concentrations were measured for all three samples, and in general no obvious concentration dependence could be detected despite slight aggregate formation at the highest concentrations of TG2-GDP and the Fab fragment. For TG2-GDP, analysis was done on data collected at the second highest concentration (5.5 mg/ml). For the Fab fragment, analysis was done on merged data from scattering profiles obtained at the lowest (2 mg/ml) and the highest (16 mg/ml) concentrations. For the complex, the analysis was performed on the data collected at the highest concentration (9 mg/ml). The overall parameters derived from the scattering profiles are summarized in Table 2. The molecular models and experimental SAXS data have been deposited on SASBDB (Small Angle Scattering Biological Data Bank; accession numbers SASDA28, SASDA38 and SASDA48). The molecular weight estimations for TG2-GDP suggested that the sample was mostly monomeric in solution. However, especially for small angles, the fitting of the experimental curve with the theoretical scattering curve of TG2-GTP (PDB code 4PYG; χ^2^ (crysol):1.5) could be significantly improved if a volume fraction of 7–9% dimeric species was taken into account (χ^2^ (Oligomer):0.9; Fig. 1).

**TABLE 2:**
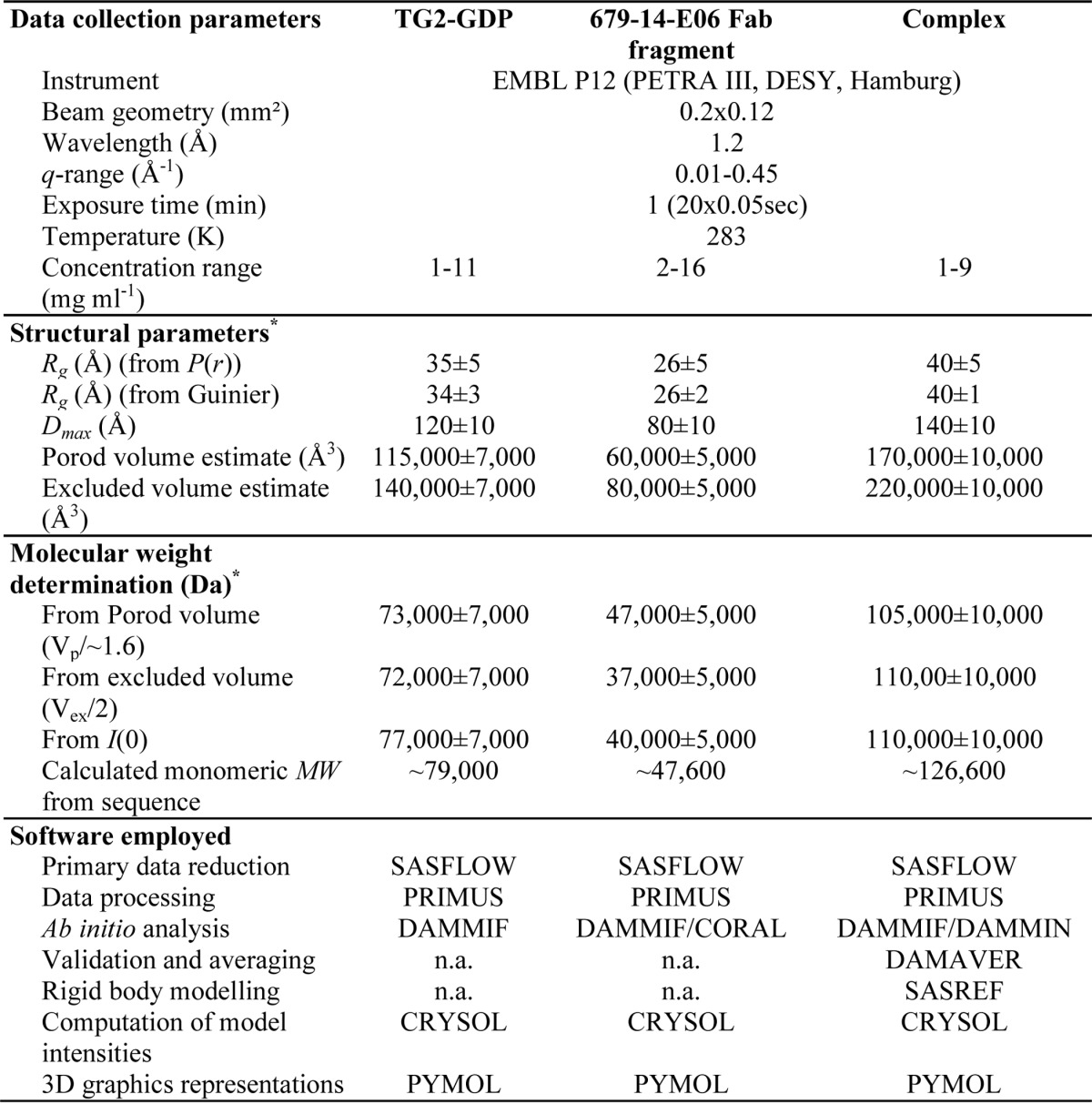
**Data collection and structure statistics for small angle x-ray scattering analysis** n.a., not applicable.

The molecular weight estimations for the Fab fragment also suggested that this subunit was monomeric in solution. Indeed, the fit of the theoretical curve based on the crystal structure fitted well to the experimental curve at low values for the scattering vector *q* but showed large discrepancy at *q* values higher than 0.1 Å^−1^ (χ^2^ (crysol): 3.6). The fit could be significantly improved by adding dummy atoms for the missing residues in the C region domains to the high resolution structure with Coral (new χ^2^ (Crysol):1.1; Fig. 1).

**FIGURE 2.**
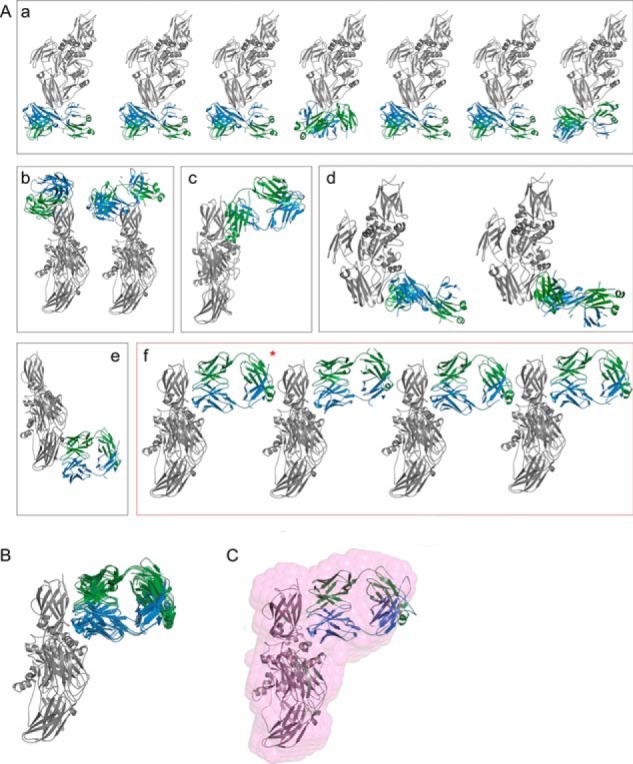
**Rigid body models of TG2-GTP in complex with 679-14-E06 Fab fragment obtained by SASREF.** *A*, the models of 17 individual rounds of SASREF were clustered into six groups (*a–f*). TG2-GTP is colored in *gray*, and the light and heavy chains of the Fab fragment are colored in *green* and *blue*, respectively. The model used for further analysis is labeled with a *red asterisk. B*, structural overlay of all models composing group *f. C*, superposition of the representative rigid body model of group *f* with the *ab initio* model for the complex (*pink beads*).

To analyze the binding site of the Fab fragment to TG2-GDP, we studied the scattering behavior of the complex in solution. *Ab initio* models as well as the molecular weight estimations suggested that the binding ratio was 1:1. Rigid body modeling was performed with SASREF to determine possible binding interfaces using the crystal structures of the Fab fragment and TG2-GTP. Altogether, 17 different models were calculated with no restricting constraints applied (Fig. 2*A*). The obtained models could be classified into six different groups (*a–f*). The generation of these different models reflects the intrinsic limitation of SAXS as a low resolution method. However, this can be overcome by combining the results with additional information collected with complementary methods. Members of group *a*, *b*, and *c* did not involve antigen-antibody interaction via the CDR loops of the Fab fragment, indicating that these do not reflect the real interaction. As epitope mapping of 679-14-E06 by hydrogen/deuterium exchange indicated that the epitope is located in the N-terminal domain of TG2 (20), the classes *d* and *e*, in which the Fab fragment associates with the C-terminal half of TG2, were also eliminated. Thus, the four models of group *f* most likely represent the real binding behavior. All of these models were highly similar with <0.7 Å normalized spatial discrepancy (Fig. 2*B*). In addition these models superimposed very well with the generated *ab initio* model (Fig. 2*C*). The fit of a selected model from group *f* to the observed data is shown in Fig. 1. Due to its higher stability with bound Fab fragment, a closed rather than an open conformation of TG2 was used to generate the complex even though TG2 is expected to adopt the open conformation in the extracellular environment. An overlay of the SASREF complex model with the x-ray structure of the open conformation (PDB code 2Q3Z) revealed that the two structures superimpose well at the binding interface. Thus open and closed TG2 should bind the same way to 679-14-E06.

##### Validation of the Small Angle X-ray Scattering Model by Site-directed Mutagenesis of TG2

**FIGURE 3.**
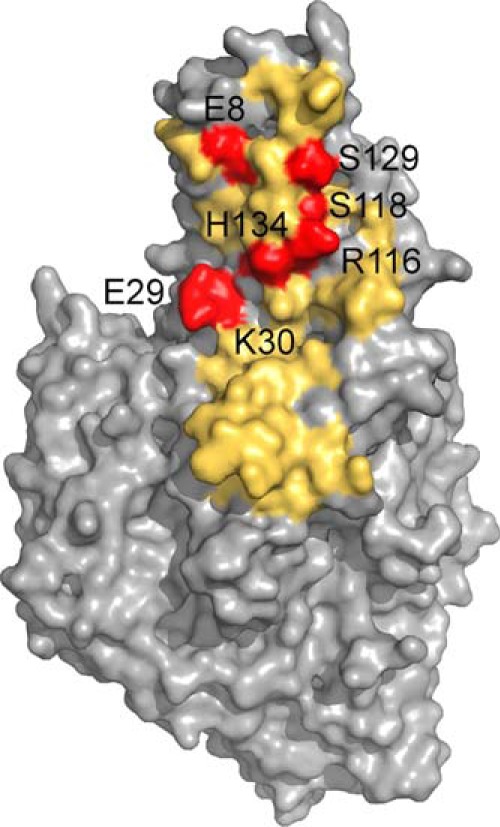
**Visualization of footprint on TG2 by the 679-14-E06 Fab fragment as indicated by SASREF.** Residues of TG2, which in the representative rigid body model of group *f* are within 5 Å distance to residues of the 679-14-E06 Fab fragment, are colored in *yellow*. Residues selected for mutagenesis analysis are colored in *red*. The residues Glu-29, Lys-30, Arg-116, Ser-118, Ser-129, and His-134 are within 5 Å distance, whereas residue Glu-8 has a 6 Å distance to the Fab fragment.

**FIGURE 4.**
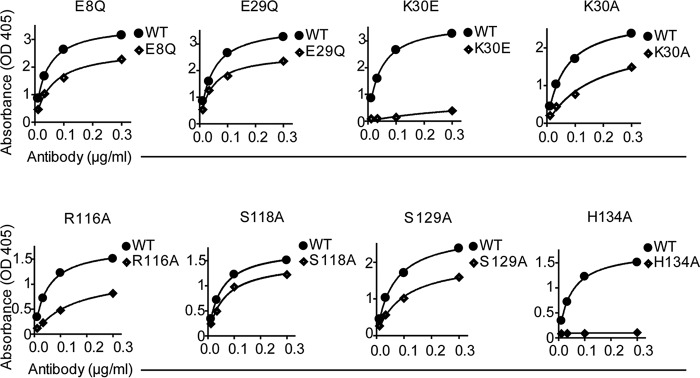
**Binding of antibody 679-14-E06 to mutants of TG2 as assessed by ELISA.** Titration curves showing binding of the antibody to the TG2 mutants E8Q, E29Q, K30E, K30A, R116A, S118A, S129A, and H134A compared of WT TG2. Experiments have been performed at least twice.

**FIGURE 5.**
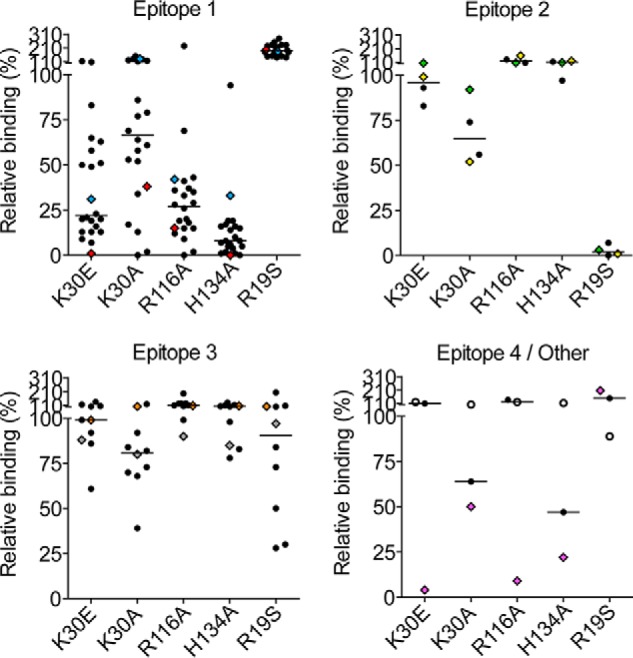
**Binding of a panel of monoclonal antibodies to TG2 mutants as assessed by ELISA.** Binding of a panel of antibodies reactive with epitope 1, 2, 3, or 4 was tested against the TG2 mutants R19S, K30A, R116A, and H134A. Shown are signals normalized against those obtained with WT TG2. The *open circles* represent an anti-TG2 antibody that was not assigned to epitope 1–4, and *diamonds* represent antibodies analyzed in the SPR study. The symbols for the two epitope 1 antibodies 679-14-E06 and 693-10-B06 are shown in *red* and *blue*, respectively. Symbols representing antibodies 693-1-A03 and 763-4-B06 recognizing epitope 2 are colored *yellow* and *green*. Symbols for the epitope 3 antibodies 763-4-A06 and 763-4-E05 are shown in *orange* and *gray*, and symbols for 693-1-D03, which recognizes epitope 4, are shown in *violet. Horizontal lines* indicate medians. Results from one of three independent experiments are shown.

In agreement with the findings that the TG2 mutations E8Q and K30E disrupt binding of epitope 1 antibodies (20), the representative rigid body model of group *f* indicated that these two residues are part of the Fab footprint on TG2 (Fig. 3). To further confirm the location of the epitope, we generated additional TG2 N-terminal domain mutants in which single amino acids predicted to interact with 679-14-E06 were changed. Thus, in addition to the two previously reported mutants, the TG2 mutants E29Q, K30A, R116A, S118A, S129A, and H134A were tested for binding by 679-14-E06 in ELISA (Fig. 4). The antibody binding to the TG2 mutants K30E, R116A, and H134A was clearly impaired. The antibody binding to the mutants E8Q, E29Q, K30A, and S129A was also reduced but to a lesser degree. The antibody binding was unaffected by the S118A mutation. A panel of 39 anti-TG2 antibodies was then tested for binding to the mutants K30A, K30E, R116A, H134A, and R19S (Fig. 5). For epitope 1 antibodies, clear effects were observed for K30E, R116A, and H134A. By contrast, antibodies targeting epitope 2 or epitope 3 were in general unaffected by these mutations. Some antibodies targeting these epitopes, however, were affected. This could be explained by overlapping footprints of these antibodies with the binding region of epitope 1, although it cannot be excluded that the mutations induced minor structural changes that could affect binding of some antibodies. Epitope 2 antibodies were affected by the R19S mutation, which is consistent with previous findings (20).

**FIGURE 6.**
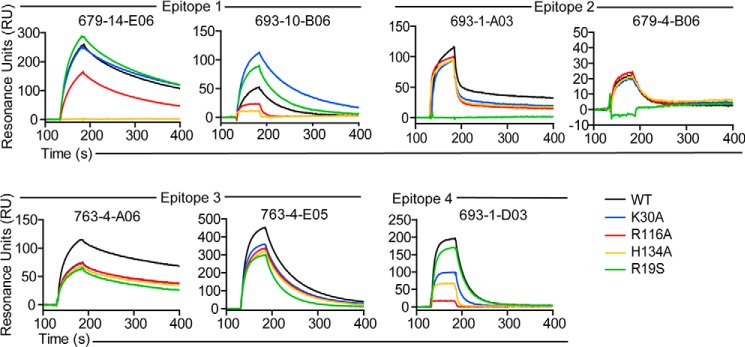
**Binding of monoclonal antibodies to mutants of TG2 as assessed by surface plasmon resonance.** The antibodies tested are representative for antibodies reactive to epitopes 1–4. The sensorgrams depict relative binding of WT and mutant TG2 variants after injection over immobilized anti-TG2 autoantibodies. Shown are representative sensorgrams from one of two experiments.

**FIGURE 7.**
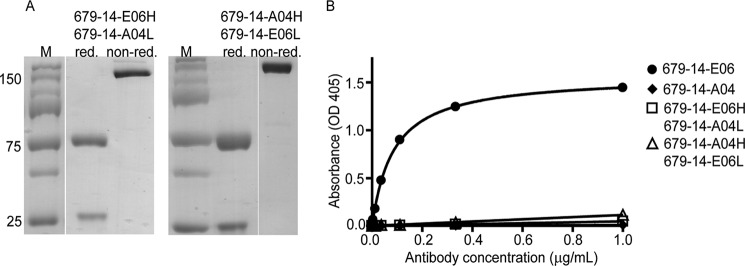
**Both heavy and light chain of 679-14-E06 are involved in binding of epitope 1.** Heavy and light chains were swapped between antibodies reactive (679-14-E06) and not reactive (679-14-A04) to TG2. *A*, Coomassie-stained SDS-PAGE gels showing 679-14-E06H/679-14-A04L and 679-14-A04H/679-14-E06L antibodies under reducing and non-reducing conditions (*white lines* show the borders of different lanes). *B*, reactivity of the antibodies 679-14-E06, 679-14-A04, 679-14-E06H/679-14-A04L, and 679-14-A04H/679-14-E06L to TG2 as monitored by ELISA. Shown are the results from one of two independent experiments.

Next, a panel of seven representative antibodies reactive with epitopes 1, 2, 3, or 4 was tested for binding to WT TG2 and the mutants R19S, K30A, R116A, and H134A by SPR (Fig. 6). The sensorgrams were fitted to a simple 1:1 Langmuir binding model, and kinetic constants were derived (Table 3). For antibody 693-1-A03, accurate kinetic parameters could not be obtained due to poor fit to the model as a result of biphasic binding (Fig. 7). Overall, the SPR results were in agreement with the ELISA results. For both epitope 1 antibodies tested, we observed impaired binding to the mutants R116A and H134A, whereas the epitope 2 and epitope 3 antibodies were unaffected by these mutations. The R116A and H134A mutations were also found to negatively affect the recognition by the epitope 4 antibody (Fig. 6). This is consistent with the ELISA results and the finding that epitope 1 and 4 partly overlap (19). The R19S mutation reduced recognition by the two epitope 2 antibodies but not to any of the other antibodies of the panel (Fig. 6). Surprisingly, the epitope 1 antibody 693-10-B06 bound the K30A mutant with increased strength compared with WT TG2 in both ELISA and SPR, although the same antibody showed lower reactivity with the K30E mutant. Similar behavior was also observed for some other epitope 1 antibodies in ELISA. This finding probably reflects unique properties of some antibodies, making the K30A mutation favorable for binding, whereas the K30E mutation disrupts the interaction. Altogether, the testing of the mutants strongly suggests that epitope 1 contains the TG2 residues Arg-116 and His-134 along with the previously reported residues Glu-8 and Lys-30 (20). In addition some, but weaker, evidence was found for residues Glu-29 and Ser-129 being involved in antibody binding.

**TABLE 3:** **Surface plasmon resonance derived binding kinetics. Data are presented as the means ± S.D. based on two experiments**

Epitope	Antibodies	Kinetic parameters	WT	R19S	K30A	R116A	H134A
1	679-14-E06	*k_a_* (10^4^/ms)	8.53 ± 0.4	3.58 ± 0.3	7.08 ± 0.9	3.06 ± 0.3	ND*^a^*
		*k_d_* (10^−3^/s)	4.74 ± 0.4	5.25 ± 0.2	3.53 ± 0.5	7.36 ± 1.3	ND
		kDa (nm)	55.5	146.6	49.9	240.5	ND
	693-10-B06	*k_a_* (10^4^/ms)	8.04 ± 0.9	7.15 ± 0.5	7.1 ± 0.9	2.71 ± 0.5	ND
		kd (10^−3^/s)	23.9 ± 1.3	18.7 ± 0.9	10.2 ± 0.6	145.0 ± 11.3	ND
		kDa (nm)	297.3	261.5	143.7	5350.5	ND
2	763-4-B06	*k_a_* (10^4^/ms)	37.8 ± 13.3	ND	32.1 ± 0.2	45.7 ± 1.8	31.3 ± 10.7
		*k_d_* (10^−3^/s)	40.8 ± 2.3	ND	36.7 ± 0.3	36.6 ± 0.1	52.6 ± 3.5
		kDa (nm)	107.4	ND	114.3	80.1	168.1
3	763-4-A06	*k_a_* (10^4^/ms)	14.2 ± 0.9	11.7 ± 1.0	12.7 ± 0.8	11.3 ± 0.9	10.8 ± 1.0
		*k_d_* (10^−3^/s)	2.16 ± 0.0	3.06 ± 0.2	2.4 ± 0.1	2.3 ± 0.1	2.19 ± 0.2
		kDa (nm)	15.2	26.2	18.9	20.4	20.3
4	693-1-D03	*k_a_* (10^4^/ms)	15.3 ± 2.1	17.9 ± 3.3	19.2 ± 3.8	NA*^b^*	NA
		*k_d_* (10^−3^/s)	37.9 ± 1.9	37.7 ± 2.2	65.2 ± 7.6	NA	NA
		kDa (nm)	247.7	210.6	339.6	NA	NA

*^a^* ND, not detectable.

*^b^* NA, not acquired due to fast binding kinetics.

##### Both Heavy and Light Chains of 679-14-E06 Are Involved in Epitope 1 Binding

The importance of heavy and light chains for binding of epitope 1 by 679-14-E06 was assessed by swapping heavy and light chains of this antibody with those of the antibody 679-14-A04, which is not reactive with TG2. Both hybrid antibodies were produced efficiently by HEK 293F cells with normal pairing of the heavy and light chains (Fig. 7*A*). By testing in ELISA, neither of the hybrid antibodies (*i.e.* 679-14-E06H/679-14-A04L or 679-14-A04H/679-14-E06L) bound TG2 as compared with the efficient binding by the 679-14-E06H/679-14-E06L antibody (Fig. 7*B*). This suggests that residues of both the heavy and the light chain of 679-14-E06 are involved in recognition of epitope 1.

##### Molecular Dynamics Simulation of the Binding of 679-14-E06 Fab Fragment to TG2

To further understand the binding of 679-14-E06 to TG2 and the effects of the distinct mutations, we performed MD simulation using a representative rigid body model of group *f* obtained by SASREF as a starting model. After 1.1 ns an equilibration state was reached for which the backbone root mean square deviation value between individual steps was <1.0 Å. Ten states were extracted from the MD trajectory in its equilibrium state. The total mean binding energy of −475 kcal/mol was calculated resulting from electrostatic (−447 kcal/mol) as well as hydrophobic van der Waals interactions (−28 kcal/mol).

##### Details of the Epitope and Paratope Predicted by the MD Simulation

**FIGURE 8.**
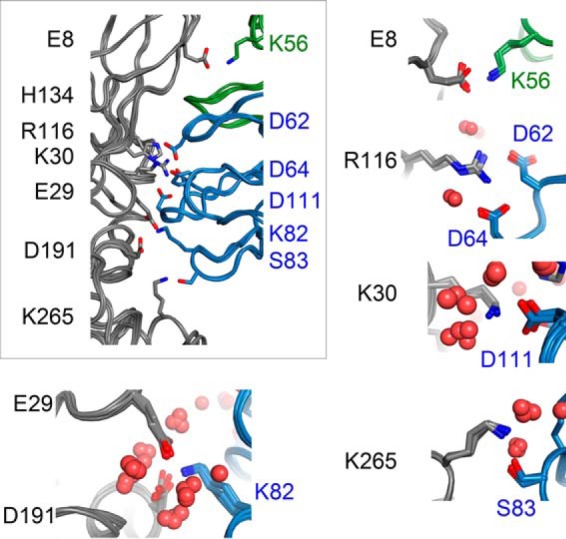
**Structure models derived from molecular dynamics simulation of the interaction between TG2 and the Fab fragment of 679-14-E06.** The ribbon overlay of the backbone for four states of the MD trajectory is shown in the *box*. The residues engaged in the binding between the Fab and TG2 of one of the states are shown as *sticks*. TG2 is colored in *gray*, whereas the light chain and heavy chain of the Fab fragment are colored in *green* and *blue*, respectively. The surrounding close-up views depict overlays of four states and show the side chains of the amino acid residues engaged in binding. *Red spheres* represent water molecules.

The residues of TG2, which in the MD simulation model are predicted to be involved in the interaction with 679-14-E06, are Glu-8, Glu-29, Lys-30, Arg-16, Asp-191, and Lys-265 (Fig. 8). The overlay of these side chains from four states of the MD trajectory shows that indeed these residues show little structural flexibility and thus form stable interactions with the Fab fragment. These residues constitute a sequentially discontinuous epitope mainly located in the N-terminal domain of TG2. Residues Asp-191 and Lys-265 are located in the catalytic domain, suggesting that epitope 1 also stretches into this domain. The model further suggests that the paratope mainly involves residues of the heavy chain. There are hydrogen bonds or salt bridges involving the CDR2 and CDR3 loops of the heavy chain as well as residues of the framework 3 region. In addition, one important contact is made to the light chain CDR2 loop.

**FIGURE 9.**
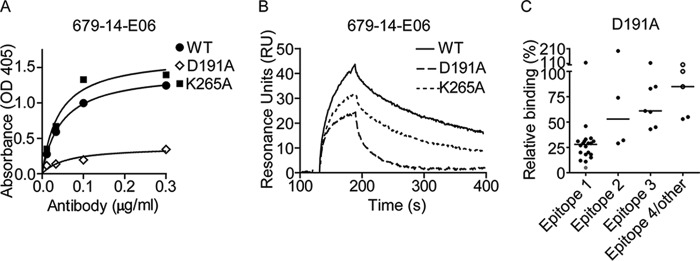
**Involvement of the TG2 residues Asp-191 and Lys-265 in the epitope of 679-14-E06 and other epitope 1 antibodies.** *A* and *B*, binding of antibody 679-14-E06 to the TG2 mutants D191A and K265A as assessed by ELISA and SPR. The ELISA and SPR experiments were done at least twice. *C*, testing of the binding to the TG2 mutant D191A by a panel of anti-TG2 antibodies reactive with epitope 1, 2, 3, or 4. Shown are signals normalized against those obtained with WT TG2. The *gray point* represents the antibody 679-14-E06, and the *open circles* represent anti-TG2 antibodies that were not assigned to epitope 1–4. *Horizontal lines* indicate medians.

As to details of the interactions with CDR loops, the model suggests that Glu-8 of TG2 forms a salt bridge with Lys-56 of CDR-L2, that Lys-30 of TG2 forms a salt bridge with Asp-111 of CDR-H3, and that Arg-116 of TG2 forms salt bridges with residues Asp-62 and Asp-64 of CDR-H2. The model also predicts interactions between residues Lys-82 and Ser-83 of the framework 3 region of the heavy chain with TG2. These residues are located in a loop that sometimes is referred to as CDR-H4 (46) and is estimated to account for 1.3% of human antibody-antigen contacts (47). Specifically, Glu-29 and Asp-191 of TG2 make salt bridges with Lys-82, and Lys-265 of TG2 forms a hydrogen bond with Ser-83 in the model. Testing of the mutants D191A and K265A in ELISA and SPR with the antibody 679-14-E06 did not confirm involvement of Lys-265 in the epitope of 679-14-E06 (see Fig. 9, *A* and *B*). There was, however, some evidence for epitope involvement of the residue Asp-191. The binding of 679-14-E06 and other epitope 1 antibodies to D191A was strongly impaired (Fig 9*C*). Albeit to a clearly lesser extent, also other antibodies targeting epitope 2 or 3 showed less binding to this mutant, precluding a firm conclusion of the involvement of this residue in epitope 1.

In addition to direct electrostatic interactions between residues of TG2 and the Fab fragment, the MD model also reveals a tightly packed water network at the epitope-paratope interface. This water network connects several residues in the binding pocket to each other. Most prominently, His-134 is involved in the well-connected water network. The residues Lys-30 and Arg-116 from TG2 as well as the residues Tyr-57 (CDR-H2), Asp-62 (CDR-H2), and Asp-111 (CDR-H3) of 679-14-E06 are also involved.

**FIGURE 10.**
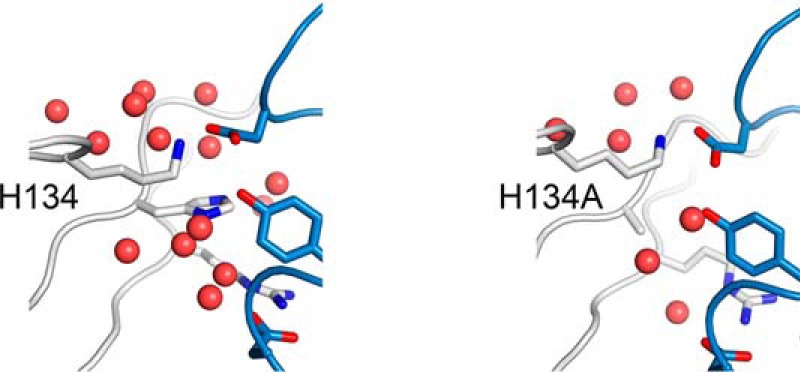
**Structure from molecular dynamics simulation revealing the involvement of the water network around residue His-134 in interaction with the heavy chain of 679-14-E06.** At the binding interface a network of water molecules surrounds the histidine residue. This water network is disrupted by replacing histidine with alanine.

To confirm the important role of His-134 in this water pocket, we performed a MD simulation with an alanine at this position (H134A). In the simulation, the substitution of histidine with alanine at position 134 of TG2 led to a disruption of the water network at the antibody binding interface (Fig. 10). The lack of water molecules leads to a stabilization of the neighboring electrostatic interactions as indicated by an increased calculated binding energy (−562 kcal/mol). However, the formation of this interface would require a transition state in which the water molecules are removed. Thus, the energetic penalty during this binding process could prevent binding in the first place. This scenario could explain the reduced binding affinity of the Fab fragment to the H134A mutant in the biochemical assays.

## Discussion

Previous studies have shown that antibodies produced by TG2-specific gut plasma cells target a few epitopes that partly overlap and cluster in the N-terminal part of TG2 (19, 20). Insights about the binding sites of these autoantibodies will be helpful to understand the basis for selection and activation of autoreactive B cells in celiac disease. In addition, knowing how the antibodies interact with TG2 should help us understand if they can modify the function of TG2 and thereby play a pathogenic role. In this study we have characterized epitope 1, which is a frequently targeted epitope by celiac disease autoantibodies. Together, the data from hydrogen/deuterium exchange (20) and SAXS/MD modeling reveal key residues of epitope 1 and also give details of the paratope of the anti-TG2 antibody 679-14-E06).

The lack of reactivity of the two hybrid antibodies sharing either the light or heavy chain with 679-14-E06 suggests that residues of both the heavy and light chain make essential contributions to antigen recognition by the antibody, although dysfunctional pairing of heavy and light chains cannot be ruled out as a reason for the failed reactivity. This notion is supported by the MD model, which suggested that interaction of 679-14-E06 with TG2 involves CDR loops of both the heavy and light chain. Strikingly, epitope 1 antibodies have biased usage of the *IGHV5-51* and *IGKV1-5* gene segments (3, 48). Involvement of CDR-L2, CDR-H2, and CDR-H3 as well as the interaction of CDR-H4 framework three residues could explain the preferential usage of heavy and light gene segments by epitope 1-specific antibodies.

The predictions of the MD model fit well with results of hydrogen/deuterium exchange experiments with antibody 679-14-E06 (20). This antibody had an effect on deuterium uptake for the TG2 peptide fragments covering residues 5–12, 27–40, 130–135, and 130–137, reflecting the involvement of Glu-8, Glu-29, Lys-30, and His-134 in antibody binding. Residues Arg-116, Asp-191, and Lys-265, which were also identified as interacting residues, were not part of the fragments identified by mass spectrometry in the previous study.

The MD model identifies residues involved in the epitope and paratope of 679-14-E06. The mutational data largely confirm involvement of residues of the epitope. Some caution should, however, be exercised on the description of the paratope as this is based on an *in silico* model. The residues most clearly identified by mutational analysis to be part of epitope 1 of TG2 are residues Arg-116 and His-134. The MD model provides an explanation for the impaired recognition of R116A mutant as this mutation will disrupt the salt bridges to Asp-62 and Asp-64 of CDR-H2. The effect of the H134A mutation can be described through the disruption of the water network around His-134 and the presumed energy penalty for the removal of these water molecules.

The R116A and H134A mutants were also less well recognized by one of two epitope 4 antibodies. This is not surprising, as epitopes 1 and 4 partly overlap (19, 20). As previously reported, the TG2 mutant R19S was poorly recognized by epitope 2 antibodies, whereas the mutations R116A and H134A did not affect binding of these antibodies. Based on competitive ELISA experiments, it was previously concluded that epitopes 1 and 2 do not overlap (19). Our results suggest that antibodies recognizing epitope 1 and epitope 2 bind at opposite sides of the N-terminal domain of TG2. Epitope 1 is located so that the active site of TG2 points toward the antibody/B-cell receptor upon binding. It was suggested that this property favors activation of epitope 1-targeting B cells by allowing TG2 to form isopeptide cross-links between B-cell receptors on the cell surface (20). This could explain why epitope 1 appears to be targeted more frequently than the other epitopes among TG2-specific gut plasma cells in celiac disease (3). The results of this study corroborate the structural basis for this idea.

In the MD model Lys-30 of TG2 makes a salt bridge with Asp-111 of CDR-H3. Involvement of Lys-30 in epitope 1 is supported by the TG2 mutation data. Notwithstanding, epitope 1 antibodies displayed different binding behavior to the two mutants K30E and K30A (Fig. 5). Although K30E has a strong impact on antibody binding (20), the K30A mutation had a minor effect. Possibly the introduction of a negative charge in the K30E mutant could create electrostatic repulsion between the antibody and TG2, thus making the antibody binding more sensitive to this mutation.

Results from testing binding of antibodies to TG2 mutants in the current and a previous study (20) suggest that residues Glu-8 and Glu-29 are part of epitope 1. The MD simulation model of the interaction of 679-14-E06 with TG2 gives credence to this notion. In the model Glu-29 of TG2 forms a salt bridge with Lys-82 of framework 3 of the heavy chain (*i.e.* CDR-H4), whereas Glu-8 forms a salt bridge with Lys-56 of CDR-L2.

The MD model predicts the involvement of residues Asp-191 and Lys-265 in the catalytic domain of TG2 in interaction with 679-14-E06. Although we were unable to confirm the involvement of Lys-265 by mutation analysis, we obtained some evidence that Asp-191 is part of the epitope of 679-14-E06 and other epitope 1 antibodies.

The MD model predicted that the S118A mutation should not impact binding, as this residue does not make direct contact with the Fab fragment of 679-14-E06. This is in agreement with mutational analysis revealing that the S118A mutation did not affect binding of 679-14-E06.

Allelic polymorphism of immunoglobulin genes may potentially contribute to susceptibility to immune-mediated diseases, particularly in diseases where recognition of particular epitopes is of importance. In line with this notion, it was recently demonstrated in mice that polymorphic residues of the heavy chain variable region dictate the antibody response to an epitope of collagen implicated in collagen-induced arthritis (33). Given the restricted usage of heavy and light gene segment usage in response to TG2 in celiac disease, immunoglobulin gene polymorphisms could potentially have an impact in this disorder. We, therefore, investigated whether any known polymorphic residues of *IGHV5-51* and *IGKV1-5* are located in regions of the MD model where 679-14-E06 interacts with TG2. For *IGHV5-51* there are three single nucleotide polymorphisms giving rise to the amino acid substitutions I39T, G47R, and S83P. None of these residues, with the possible exception of Ser-83, which has an experimentally non-confirmed interaction with Lys-265 in our MD model, makes contact with TG2. For *IGKV1-5*, a particularly interesting residue is Lys-56, which is located in CDR-L2. There are two major *IGKV1-5* alleles displaying variance at this position. Allele 3, used by 679-14-E06, carries lysine, whereas allele 1 carries aspartate. In the MD model, Lys-56 forms a salt bridge with Glu-8 of TG2. Aspartate at position 56 would most likely result in repulsion and prevent the binding of the antibody to TG2. All antibodies using *IGKV1-5* in the limited panel of antibodies established from four individuals do carry Lys-56 (3), but this is also the most common of the two alleles among Europeans (SNP frequency ∼90% according to the NCBI dbSNP database). Thus, further analysis is required to address whether this polymorphism may dictate the antibody response to TG2 in celiac disease.

## Author Contributions

X. C., K. H., M. A. G., R. I., A. T., and L. M. S. designed the study and wrote the paper. X. C. purified and crystallized the Fab fragment, determined its x-ray structure and prepared the samples for the SAXS experiments. K. H. cloned the Fab fragment, purified the antibodies, and performed the mutagenesis analysis, ELISA, and SPR studies. X. C. and K. H. produced and tested the antibodies with swapped heavy and light chains. X. C. and K. H. also performed the SAXS experiments under the supervision of M. A. G. M. A. G. and D. S. analyzed the SAXS data. J. T. A. helped designing the SPR experiments. R. I. performed part of the ELISA experiments. A. T. performed the MD. All authors analyzed the results and approved the final version of the manuscript.

## Footnotes

4 The abbreviations used are:

TG2: transglutaminase 2
SAXS: small angle x-ray scattering
CDR: complementarity determining region
SPR: surface plasmon resonance
RG: radius of gyration
MW: molecular weight
RU: resonance units
MD: molecular dynamics.
